# Poly(*O*‐Propargyl‐*N*‐Amino Carbamate), a Reactive Polymer to Underpin Biomedical Applications of Poly(acetylene)s

**DOI:** 10.1002/marc.202500566

**Published:** 2025-10-25

**Authors:** Tom Leigh, Greta Bellio, Daniel Crisan, Amit Deb, Alba Ramil‐Bouzas, Andrey Romanyuk, Ivan Torreiro‐Leon, Ana Rey‐Rico, Paco Fernandez‐Trillo

**Affiliations:** ^1^ School of Chemistry University of Birmingham Birmingham UK; ^2^ BioNanoChem Lab Centro Interdisciplinar de Química e Bioloxía – CICA Universidade Da Coruña A Coruña Spain; ^3^ G‐Cel, Centro Interdisciplinar de Química e Bioloxía – CICA Universidade Da Coruña A Coruña Spain; ^4^ Facultade De Ciencias Universidade Da Coruña A Coruña Spain

**Keywords:** biocompatible polymers, poly(acetylene)s, post‐polymerization modification

## Abstract

Synthetic polymers are widely used in biomedical applications, yet most lack defined secondary structures common in nature, such as helices or β‐sheets. In particular, dynamic helical polymers such as poly(acetylene)s have been rarely explored in this field. Here, we report the preparation of a new reactive poly(acetylene), poly(*O*‐propargyl‐*N*‐amino carbamate) (**P1**), as a platform for the preparation of functional poly(acetylene)s in water. **P1** was prepared from readily available starting materials through Rh‐catalyzed polymerization and acid‐mediated deprotection. ^1^H‐NMR indicated that the formed polymer had a high content of *cis* double bonds, while UV–vis and DSC suggested a predominant *cis*‐*cisoid* conformation. This conformation was also observed via CD for chiral derivatives of Boc‐protected **P1**, while a partially deprotected chiral derivative of **P1** could be adopting multiple conformations in solution, including *cis*‐*transoid* and *cis*‐*cisoid* conformations. **P1** was functionalized under aqueous conditions with a small set of aldehydes, including cationic, aliphatic, and aromatic derivatives, as well as a small set of carbohydrates. Finally, we demonstrate that **P1**, and a guanidinium derivative **P1‐*mod*‐4**, were well tolerated by cells, highlighting the potential of this polymer to underpin the development of biomedical applications of poly(acetylene)s.

## Graphical abstract

We report here a new methodology to prepare functional poly(acetylene)s under aqueous conditions. This methodology is underpinned by poly(O‐propargyl‐N‐amino carbamate) (**P1**), a reactive poly(acetylene) carrying acyl hydrazines. Thus, **P1** reacts with aldehydes to give functional poly(acetylene)s, including cationic, hydrophobic, and sugar‐loaded poly(acetylene)s. Moreover, we demonstrate that **P1** and a cationic derivative are non‐toxic to a small panel of cell lines.

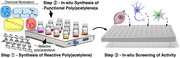

## 1 Introduction

Synthetic polymers have long been explored in biomedical applications. Since the early adoption of poly(methyl methacrylate) in contact lenses or the application of nylon for sutures, both in the 1940s, the use of polymers in biomedicine has only continued to grow [1]. More importantly, the role of these polymers has continued to evolve over this time. While most of the early applications of synthetic polymers used them as structural materials, in implants, catheters, and other devices, more and more applications rely on these materials having an “active” role [1]. Synthetic polymers like poly(ethylene glycol) are now commonly used in clinical settings to give drugs stealth properties (i.e., shield the drug from degradation and increase circulation times) [2, 3]. Others, such as poly(*N*‐isopropylacrylamide), confer drugs, nanocarriers, and devices with thermoresponsive properties, so that the shape, topology, or solubility of the system changes with changes in temperature [4]. Polymers that respond in a similar fashion to other stimuli, such as pH, the application of an external source of light, or the presence of specific enzymes in the body, have also been developed [5]. And while the range of polymers with intrinsic therapeutic potential is still limited, polymers like Sevelamer [6, 7] or Colesevelam [8] are now being used in clinical settings as scavengers of misregulated metabolites. Antimicrobials [9, 10], or inhibitors of protein–protein interactions [11] are other areas where the potential of synthetic polymers as therapeutics has been identified.

Most of the polymers used so far in biomedical applications have a random secondary structure, with polymer backbones that do not adopt any particular conformation. This is surprising because, while there are many biomolecules and biopolymers that do not have a defined secondary structure, such as mucins [12, 13] or intrinsically disordered proteins [12, 14], most proteins, nucleic acids, and carbohydrates adopt a preferred secondary structure that is critical for their biological activity. Helices are probably the most prevalent of these secondary structures, being integral for the storage of genetic information [15], the formation of connective tissue [16, 17], or the translocation across biological barriers [18, 19]. Despite this relevance, the application of helical polymers in biomedicine is still in its infancy [20, 21]. Most of these applications have focused on helical poly(amino acid)s that not only mimic the secondary structure of natural helices, but also rely on the same building blocks (i.e., amino acids). Using natural building blocks should lead to biocompatible materials, although non‐native peptides and poly(amino acid)s can trigger negative immune responses [22, 23]. Moreover, the secondary structures obtained are mainly reduced to those observed for the natural materials, which can limit the range of applications for these “bio‐based” polymers. Much less used are other helical polymers such as poly(isocyanide)s or poly(acetylene)s, despite their potential to give helical materials with unique properties [20, 21]. The lack of applications of poly(acetylene)s is particularly surprising, as these dynamic helical polymers can adapt their helicity in aqueous conditions to external stimuli [24, 25, 26] such as pH [27, 28, 29, 30, 31, 32, 33, 34, 35, 36, 37], temperature [38, 39, 40, 41, 42, 43], or the presence of relevant metabolites, such as glucose [44], amino acids [32, 33, 45], other biorelevant molecules [27, 44, 46, 47, 48], or metals, including essential elements [37, 49, 50, 51, 52]. This dynamic helicity is particularly attractive for the development of smart materials that can change their shape, topology, or solubility in response to changes in the environment [20, 21]. Although poly(acetylene)s can be prepared with good control in the presence of air and moisture [53, 54, 55, 56], most poly(acetylene)s investigated so far have been prepared in organic solvents and give polymers that are insoluble in water [53, 54, 55, 56]. This lack of solubility is not surprising because packing around a helix can promote intramolecular hydrogen bonding between the side chains, limiting the access of solvent and thus the solubility of the helices. The lack of solubility of polymers following the adoption of a helical topology was early illustrated for poly(amino acid)s such as poly(glutamic acid), which will precipitate out of solution as its charges were neutralized [57, 58]. Solubility can be improved by increasing the length of the side‐chains to stabilize the helical topology while providing flexibility to the side‐chains to accommodate solvent upon solvation [20, 21].

**SCHEME 1:**
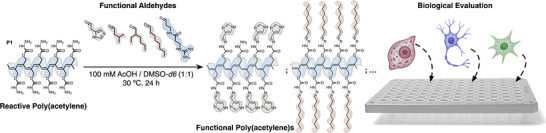
Poly(*O*‐propargyl‐*N*‐amino carbamate) (**P1**) and its post‐polymerization functionalization, under aqueous conditions, with functional aldehydes, to give a small library of functional poly(acetylene)s that can be evaluated in situ in biomedical applications.

We report here the synthesis of a new reactive polymer, poly(*O*‐propargyl‐*N*‐amino carbamate) (**P1**), as a scaffold for the preparation of functional poly(acetylene)s in aqueous conditions (Scheme 1). **P1** was prepared using a short synthetic procedure that includes the polymerization of a protected monomer in the presence of [Rh(nbd)BPh_4_], and deprotection under mild conditions to avoid degradation of the polymer backbone. Using NMR, we demonstrate that **P1** had a high content of *cis* double bonds while its UV–vis spectra and DSC were consistent with a predominant *cis‐cisoid* conformation. A CD of chiral derivatives of the protected polymer was employed to demonstrate the presence of this preferred conformation and indicated that a partially deprotected **P1** could be adopting multiple conformations in aqueous solutions, including the helical *cis‐cisoid* and *cis‐transoid* conformations. The presence of acylhydrazine moieties in **P1** side‐chains allowed us to quickly access a small library of functional polymers through the reaction of **P1** with aldehydes and saccharides in aqueous conditions. Our data indicate that the degree of functionalization depended on the ability of these aldehydes to form non‐covalent interactions such as H‐bonds and Van der Waals interactions, suggesting a potential stabilization of the helical conformation upon functionalization. Finally, we demonstrate that both **P1** and a cationic derivative were well tolerated by cells, paving the way for this reactive polymer to underpin biomedical applications of poly(acetylene)s.

## 2 Materials and Methods

### 2.1 Materials

[Rh(nbd)BPh_4_] [59] and 3‐guanidino‐*N*‐(3‐oxopropyl)propanamide (**4**) [60] were synthesized according to protocols described in the literature. Cell Counting Kit‐8 (MedChemExpress, NJ, US) was purchased from CliniSciences. All other chemicals were purchased from commercial suppliers and used without further purification. All solvents were Reagent grade or above, purchased from commercial suppliers, and used without further purification.

### 2.2 Equipment

Nuclear Magnetic Resonance (NMR) spectra were recorded on either a Bruker Avance III 300 MHz or a Bruker Avance III 400 MHz spectrometer. Chemical shifts are reported in ppm and referenced to the solvent signals. Dialysis was carried out in deionized water at room temperature for a minimum of 48 h using a Spectra/Por 6 1000 Molecular weight cut‐off 38 mm width membrane. Infrared (IR) spectra were recorded on a PerkinElmer Spectrum Two FT‐IR spectrometer. Ultraviolet–visible (UV‐vis) spectra were recorded on a Cary 50 Spectrophotometer. Gel Permeation Chromatography (GPC) was performed with a Shimadzu Prominence LC‐20A fitted with a Thermo Fisher Refractomax 521 Detector and a SPD20A UV‐vis Detector. Boc‐Protected polymers were analyzed using 0.05 M LiBr in dimethylformamide (DMF) at 60°C, or 0.005 M NH_4_BF_4_ in DMF at 50°C, as the eluent and a flow rate of 1 mL·min^−1^. The instrument was fitted with a Polymer Labs PolarGel guard column (50 × 7.5 mm, 5 µm) followed by two PLGel PL1110–6540 columns (300 × 7.5 mm, 5 µm). Molecular weights were calculated based on a standard calibration method using poly(methyl methacrylate). CD spectra were recorded on a Jasco FP‐8500, in a Quartz 400 µL cuvette with a path length of 1 mm. Dynamic Scanning Calorimetry (DSC) was recorded on the Mettler Toledo DSC 3+ STAR system. Cell viability was monitored by measuring absorbance at 450 nm using a Synergy HTX Plate Reader (Biotek, USA).

### 2.3 Synthesis of Poly(*O*‐Propargyl‐*N*‐Amino Carbamate) P1 and Chiral Derivatives

#### 2.3.1 *O*‐1‐Propargyl‐*N*‐*Tert*‐Butylamino Carbamate (**M1**)

Propargyl chloroformate (**1**) (531 mg, 4.67 mmol) was dissolved in ethyl acetate (10 mL) and cooled in ice. *tert*‐Butyl carbazate (**2**) (679 mg, 5.1 mmol) was dissolved in ethyl acetate (10 mL). This solution was added to the solution of **1** over 10 min, while stirring. The reaction mixture was then kept at room temperature for 90 min. Next, a saturated aqueous solution of Na_2_CO_3_ was added until the pH was neutralized (∼pH 7). The organic layer was then washed with 0.1 M HCl (3 × 30 mL), saturated Na_2_CO_3_(aq) (3 × 30 mL), deionized H_2_O (3 × 30 mL), and brine (3 × 30 mL). The organic layer was dried over dry Na_2_SO_4_ and filtered. The solvent was then removed under reduced pressure to give the crude product (**M1**) as a white powder (500 mg, 50% yield).

**^1^H NMR (400 MHz, CDCl_3_) / ppm**: 6.52 (s, 1H, H─N), 6.30 (s, 1H, H─N), 4.79 (d, 2H, *J* = 2.5 Hz, CH_2_), 2.52 (t, 1H, *J* = 2.5 Hz, H─C≡), 1.50 (s, 9H, CH_3_). **^13^C NMR (400 MHz, CDCl3) / ppm**: 155.69 (O═C), 81.87 (─C≡), 75.15 (≡C─H), 53.32 (CH_2_O), 27.93 (CCH3). **FTIR ν_max_ / cm^−1^**: 3279 (s, sh, H─C≡), 3024 (w, sh, H─N), 2979 (w, sh, H─C), 2136 (w, sh, C≡C), 1752 (m, sh, C═O), 1730 (s, sh, C═O), 1692 (s, sh, N─H). **UV–vis λ_max_ / nm (acetonitrile)**: 273.

#### 2.3.2 Poly(*O*‐1‐Propargyl‐*N*‐*Tert*‐Butylamino Carbamate) (Boc‐**P1**)

**M1** (1.00 g, 4.67 mmol) and [Rh(nbd)BPh_4_] (24 mg, 0.0467 mmol) were dissolved in THF (15.7 mL), and the mixture was stirred at 30°C for 2.5 h. The reaction mixture was then precipitated over excess *n*‐hexane. Next, the resulting precipitate was dried on a rotary evaporator to produce the title polymer as a pale‐yellow powder (610 mg, 61% yield).

**^1^H NMR (400 MHz, DMSO‐*d6*) / ppm**: 8.87 (s, 1H, H─N), 8.61 (s, 1H, H─N), 6.35 (s, 1H, H─C═), 4.66 (s, 2H, CH_2_), 1.38 (s, 9H, CH_3_). **^13^C NMR (400 MHz, DMSO‐*d6*) / ppm**: 155.77 (O═C), 155.63 (O═C), 79.40 (C═C), 77.56 (C═C), 52.09 (CH_2_), 28.05 (CH_3_). **FTIR ν_max_ / cm^−1^**: 3290 (m, H─N), 2979 (w, sh, H─C), 1707 (s, sh, C═O). **UV–vis λ_max_ / nm (acetonitrile)**: 290. **GPC**: Mn = 20115 g·mol^−1^; Ð = 1.41.

#### 2.3.3 Poly(*O*‐1‐Propargyl‐*N*‐Amino Carbamate) (**P1**)

Boc‐**P1** (1.00 g, 60.7 mmol) was dissolved in 10 mL of trifluoroacetic acid, and the resulting mixture was stirred for 2 h at room temperature. Excess of trifluoroacetic acid was blown off with a steady stream of Argon. 10 mL of deionized water was then added to the reaction vessel, and the solution was neutralized with a saturated aqueous solution of NHCO_3_ until pH 5–6. The polymer was then dialysed against a 100 mm aqueous solution of acetic acid and freeze‐dried to yield the title compound as a pale‐yellow powder (465.3 mg, 46.53% yield) that was stored under vacuum.

**^1^H NMR (400 MHz, DMSO‐*d6*) / ppm**: 8.82 (broad s, 1H, H─N), 8.51 (broad s, 1H, H─N), 6.35 (broad s, 1H, H─C═), 4.65 (broad s, 2H, CH_2_), 1.38 (s, >3H, CH_3_ acetate). **FTIR ν_max_ / cm^−1^**: 3292 (m, br, H─N), 2950 (w, br, H─C), 1690 (s, br, C═O), 1616 (w, br, C═O). **UV‐vis / nm (HCl)**: λ_max_ = 265. **DSC / °C**: 139, 205

#### 2.3.4 *(S)‐O*‐1‐Methylpropargyl‐*N*‐*Tert*‐Butylamino Carbamate (S‐**M1**)

*(S)*‐3‐Butyn‐2‐ol was dissolved in ethyl acetate (10 mL). 1,1’‐carbonyldiimidazole (665 mg, 5.13 mmol) was also dissolved in ethyl acetate (10 mL). The two solutions were combined, and the mixture was stirred at 60°C for 4 h. A solution of *tert*‐butyl carbazate (925 mg, 7.00 mmol) in ethyl acetate (10 mL) was then added to the reaction mixture, and the reaction mixture was left to react for another 2.5 h at 60°C and 2 h at room temperature. The organic layer was concentrated under reduced pressure, then suspended again in ethyl acetate (20 mL). The organic layer was washed with deionized H_2_O (9 × 20 mL) and 0.1 M HCl (3 × 20 mL). The solvent was then removed on a rotary evaporator, and the title compound was obtained as a white powder (500 mg, 47% yield).

**^1^H NMR (400 MHz, CDCl_3_) / ppm**: 6.51 (s, 1H, H─N), 6.32 (s, 1H, H─N), 5.44 (dq, 1H, *J* = 6.7, 2.1 Hz, CH_3_), 2.48 (d, 1H, *J* = 2.1 Hz, H─C≡), 1.53 (d, 3H, *J* = 6.7 Hz, CH), 1.47 (s, 9H, CH_3_ *^t^* Bu). **^13^C NMR (400 MHz, CDCl_3_) / ppm**: 155.65 (C═O), 81.86 (C≡), 73.57 (≡CH), 62.23 (CH), 28.27 (CH_3_ *^t^* Bu), 21.55 (CH_3_). **FTIR ν_max_ / cm^−1^**: 3371 (s, sh, H─C), 3287 (w, sh, H─C≡), 3242 (w, sh, H─C), 2988 (w, sh, H─N), 2122 (w, sh, C≡C), 1744 (s, sh, C═O), 1686 (s, sh, N‐H); **UV–vis / nm (CDCl_3_)**: λ_max_ = 280 nm.

#### 2.3.5 Poly[*(S)‐O*‐1‐Methylpropargyl‐*N*‐*Tert*‐Butylamino Carbamate] (S‐Boc‐**P1**)

The title compound was prepared from S‐**M1** following the same protocol used for the preparation of Boc‐**P1**.

**^1^H NMR (400 MHz, DMSO‐*d6*) / ppm**: 8.64 (s, 2H H─N), 6.39 (s, 1H, H─C═), 5.53 (s, 1H, CH_2_), 1.37 (s, 9H, CH_3_). **^13^C NMR (400 MHz, DMSO‐*d6*) / ppm**: 155.56 (C═O), 79.18 (C═), 67.03 (═CH), 62.03 (CH_2_), 28.05 (CH_3_), 25.51 (CH_3_ *^t^* Bu). **FTIR v_max_ /cm^−1^**: 3653 (m, br), 3265 (w, br, H─N), 2970 (s, sh, H─C), 2899 (w, sh, H─C), 1706 (s, sh, C═O). **UV–vis / nm (DMSO)**: λ_max_ = 334 nm. **GPC**: Mn = 78413 g·mol^−1^; Ð = 1.79. **CD (ACN) / mdeg @ nm**: 70 @ 302.

#### 2.3.6 *(R)‐O*‐1‐Methylpropargyl‐*N*‐*Tert*‐Butylamino Carbamate (R‐**M1**)

The title compound was prepared from *(R)*‐3‐butyn‐2‐ol following the same protocol used for the preparation of S‐**M1**.

**^1^H NMR (400 MHz, CDCl_3_) / ppm**: 6.50 (s, 1H, H─N), 6.31 (s, 1H, H─N), 5.44 (dq, 1H, *J* = 6.7, 2.1 Hz, CH_3_), 2.48 (d, 1H, *J* = 2.1 Hz, H─C≡), 1.53 (d, 3H, *J* = 6.7 Hz, CH), 1.47 (s, 9H, CH_3_ *^t^* Bu). **^13^C NMR (400 MHz, CDCl_3_) / ppm**: 155.67 (C═O), 81.86 (C≡), 73.58 (≡CH), 62.24 (CH), 28.27 (CH_3_ *^t^* Bu), 21.55 (CH_3_). **FTIR ν_max_ / cm^−1^**: 3371 (s, sh, H─C), 3290 (w, sh, H─C≡), 3242 (w, sh, H─C), 2988 (w, sh, H─N), 2122 (w, sh, C≡C), 1744 (s, sh, C═O), 1686 (s, sh, N─H); **UV–vis / nm (CDCl_3_)**: λ_max_ = 280 nm.

#### 2.3.7 Poly[*(R)‐O*‐1‐Methylpropargyl‐*N*‐*Tert*‐Butylamino Carbamate] (R‐Boc‐**P1**)

The title compound was prepared from R‐**M1** following the same protocol used for the preparation of Boc‐**P1**.

**^1^H NMR (400 MHz, DMSO‐*d6*) / ppm**: 8.63 (s, 2H H─N), 6.38 (s, 1H, H─C═), 5.53 (s, 1H, CH_2_), 1.34 (s, 9H, CH_3_). **^13^C NMR (400 MHz, DMSO‐*d6*) / ppm**: 155.61 (C═O), 79.19 (C═), 67.04 (═CH), 62.03 (CH_2_), 28.05 (CH_3_), 25.15 (CH_3_ *^t^* Bu). **FTIR v_max_ /cm^−1^**: 3658 (m, br), 3271 (w, br, H─N), 2971 (s, sh, H─C), 2900 (w, sh, H‐C), 1702 (s, sh, C═O). **UV–vis / nm (DMSO)**: λ_max_ = 334 nm. **GPC**: Mn = 105202 g·mol^−1^; Ð = 2,08. **CD (ACN) / mdeg @ nm**: −75 @ 302

### 2.4 Calculation of Monomer Conversion

Monomer conversion was calculated from the ^1^H‐NMR spectra in DMSO‐*d6* by comparing the integration of the alkynyl signals (3.4–3.6 ppm) to those of the amides (8.2–9.2 ppm). Relaxation time (T1) was set to 5 s, and the integral for the amide region was set to 2 for all spectra.

$$Monomer\;conversion\;\left(\rho \right)\;/\;\%=\frac{\equiv C-H\;Integral\;@\;t}{\equiv C-H\;Integral\;@\;t0}\;\times \;100$$

### 2.5 Calculation of Percentage of Cis Double Bonds

The percentage of *cis* double bonds in the formed polymer was calculated from the ^1^H‐NMR spectra in DMSO‐*d6* by comparing the integration of the vinyl signals (6.0–6.7 ppm) to those of the amides (8.2–9.2 ppm). Relaxation time (T1) was set to 5 s, and the integral for the amide region was set to 2 for all spectra. Alternatively, spectra could be normalized to the propargyl signals (4.0–5.2 ppm), which were also set to 2 for all spectra.

$$\%\;cis\;double\;bonds=\frac{=C-H\;Integral}{\frac{N-H\;Integral}{2}}\;\times \;100$$

### 2.6 Conjugation of Poly(*O*‐1‐Propargyl‐*N*‐Amino Carbamate) P1 with Aldehydes

In a typical experiment, 10 mg of **P1** were dissolved in a 200 mm acetic acid solution in D_2_O (500 µL). The aldehyde (2 equiv. per hydrazide) was dissolved in 1 mL of D_2_O or DMSO‐*d6*. 500 µL of the aldehyde solution was added to the polymer solution, while 500 µL of a 200 mm solution of acetic acid in D_2_O was added to the remaining aldehyde. This aldehyde solution was used as a control. The reaction was run for 24 h at 30°C. For each solution, 50 µL was removed and added to 600 µL of D_2_O or DMSO‐*d6*. 50 µL of a 57 mm solution of dimethyl sulfide in D_2_O was added as an internal standard. The relaxation time (T1) was set to 5 s, and the integral for the dimethyl sulfide was set to 6 for all samples.

$$\begin{array}{ccc} & & Degree\;of\;functionalization\;/\;\%\;\\ & & \;=\frac{CHO\;Integral\;\left(P1+Aldehyde\right)}{CHO\;Integral\left(Aldehyde\right)}\times 100 \end{array}$$

### 2.7 Assessment of Cell Viability

Cells (HeLa, HEK 293T, or iMSC#9) were seeded in 96‐well plates at an initial density of 10^4^ cells per well in DMEM with 10% fetal bovine serum and 1% penicillin/streptomycin and allowed to adhere for 24 h at 37°C. Cells were then incubated with different concentrations of **P1**. Another set of cells was incubated with **P1‐*mod*‐4,** while a third set of cells was incubated with the same concentration of **4** used to functionalize **P1** for the preparation of **P1‐*mod*‐4**. Negative and positive controls included untreated cells and cells incubated in the presence of a 0.5% aqueous solution of Triton X‐100, respectively. Cells were incubated for 24 h at 37°C and 5% CO_2_. The viability of the cell lines was then monitored using the commercial reagent Cell Counting Kit‐8, following the manufacturer's protocol.

## 3 Results and Discussion

### 3.1 Polymer Preparation and Characterization

**FIGURE 1:**
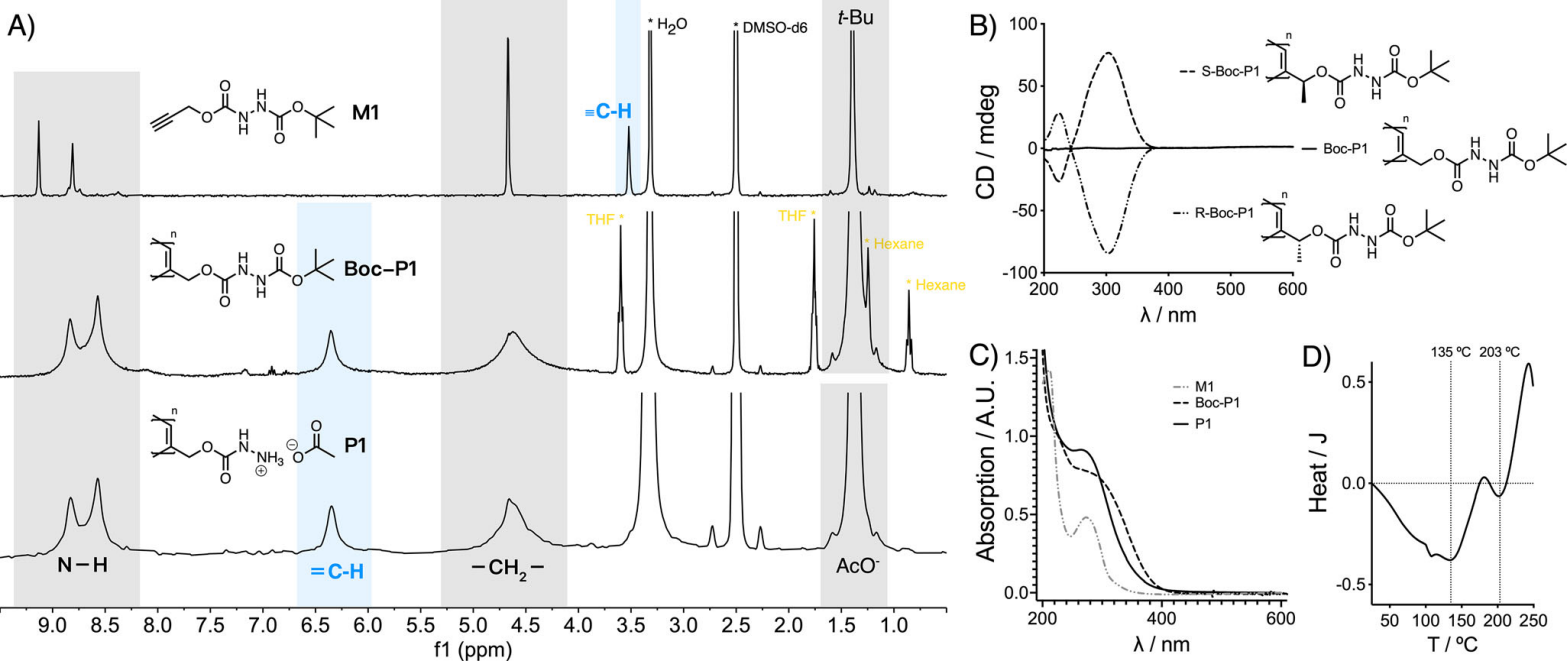
(A) Representative ^1^H‐NMR spectra of **M1** (top), Boc‐**P1** (middle), and **P1** (bottom) in DMSO‐*d6*. (B) CD spectra of S‐Boc‐**P1** (top), Boc‐**P1** (middle), and R‐Boc‐**P1** (bottom) in acetonitrile. (C) UV–vis spectra of **M1** and Boc‐**P1** in acetonitrile, and **P1** in a 100 mm aqueous solution of acetic acid. (D) DSC curve for **P1** (Heating rate 5°C·min^−1^).

Following our previous work on the application of poly(acryloyl hydrazide) [61, 62] as a reactive scaffold for the in situ synthesis and screening of polymers for gene delivery [60, 63, 64] and biocatalysis [65], our initial attempts to prepare a reactive poly(acetylene) focused on the preparation of homologous poly(propiolyl hydrazide) (Figure S1, **P0**). Unfortunately, the conjugation of the acylhydrazine moiety and the triple bond meant that this monomer was very reactive toward 1,4‐conjugate addition, leading to oligomers with the wrong polymer backbone. Commercially available propargyl chloroformate (**1**) was used instead as the starting material, thus separating the alkyne moiety from the acylhydrazine. **1** was reacted with *tert*‐butyl carbazate (**2**) to give the targeted monomer (**M1**) in moderate yields (Figure S2). Polymerization of **M1** was done with [Rh(nbd)BPh_4_], which is particularly suitable for the polymerization of propargyl amides and esters to give stereoregular polyacetylenes with high *cis* content [53, 54, 55]. Of the four stereoisomers of poly(acetylene)s, *cis‐transoid* and *cis‐cisoid* poly(acetylene)s are expected to form helices [20, 66]. Polymerization kinetics at 100 mg·mL^−1^ in THF revealed that after 1 h of polymerization ∼50% of the monomer had been consumed with the characteristic signal for the ═C─H observed at 6.4 ppm (Figure S3; Figure 1A, middle). This acetylenic proton has been observed at similar displacements for related polymers such as poly(propargyl ester)s [67, 68] and other poly(propargyl carbamate)s [69], and has been assigned to the presence of a cis double bond. In our polymerization, no additional conversion of the monomer was observed at longer polymerization times. Moreover, the signal at 6.4 ppm disappeared (Figure S3), suggesting the *cis* double bond was being degraded, potentially through isomerization to the more stable but less soluble *trans* double bond. This disappearance of the *cis* double bond was accompanied by the broadening of the NH signals at 8.6 and 8.8 ppm, suggesting that the polymer was becoming more insoluble and less ordered as the polymerization progressed. Optimization of the polymerization conditions meant that 120 mg·mL^−1^ solutions of **M1** in THF heated, in the presence of 1% of [Rh(nbd)BPh_4_], at 30°C for 2 h achieved conversions of monomer over 95% with the content of *cis* double bonds in Boc‐**P1** remaining over 70%. The targeted poly(acetylene) poly(*O*‐propargyl‐*N*‐amino carbamate) (**P1**) was obtained following deprotection with trifluoroacetic acid for approximately 2 h, and neutralization with NaHCO_3_, to avoid degradation of **P1** under strong acidic conditions. **P1** was purified as an acetate salt, following dialysis with 100 mm acetate buffer to maintain the pH at 3. As before, the presence of *cis* double bonds was confirmed by the presence of a singlet at 6.25 ppm (Figure 1A, bottom), and the content of *cis* double bonds in **P1** could be maintained over 80% following purification.

Helicity in poly(acetylene)s is associated with the appearance of a band in the circular dichroism (CD) spectra at 300–400 nm, as a result of the preferential arrangement of the helical backbone [53]. However, in the absence of any chiral bias (e.g., chiral monomer), there is no preference for a right‐ or left‐handed helix, and therefore no signal in the CD spectra. This lack of signal was the case for the prepared Boc‐**P1** (Figure 1B, solid black line) and **P1** (Figure S4, solid black line). To ensure that a preferential helical sense was observed, chiral monomers *(R)*‐*O*‐1‐methylpropargyl‐*N*‐*tert*‐butylamino carbamate (R‐**M1**) and *(S)*‐*O*‐1‐methylpropargyl‐*N*‐*tert*‐butylamino carbamate (S‐**M1**) were prepared from the corresponding chiral amines (Figure S2) and polymerized under the same conditions identified above. A clear signal at 305 nm corresponding to the polymer backbone was now observed in the CD spectra in acetonitrile for R‐Boc‐**P1** and S‐Boc‐**P1**, with both polymers having opposite directions for these bands, as expected (Figure 1B, dashed lines). Cotton effects at this wavelength have been observed for other poly(*O*‐propargyl carbamate)s [69, 70, 71] and have been assigned to polymer chains with a preferential *cis‐cisoid* conformation [71]. Full deprotection of R‐Boc‐**P1** and S‐Boc‐**P1** was not possible, as the resulting polymer quickly degraded under acidic conditions. However, we managed to isolate R‐P1* with 65% of the Boc groups cleaved. The resulting polymer was soluble (25–1.6 µg·mL^−1^) in an aqueous solution of 100 mm acetic acid at pH 3. Under these conditions, a broad positive band between 240 and 370 nm was observed (Figure S4, dashed line), suggesting that the helical topology could be preserved in aqueous conditions following this deprotection. However, multiple conformations were probably present, including random coils, and polymers with a *cis‐transoid* backbone that tend to absorb at higher wavelengths in the UV‐vis as a result of the extended conjugation of the system [72, 73].

Further characterization of **P1** and its precursors was done via UV‐vis spectroscopy. The UV‐vis spectra of the starting monomer **M1** showed a weakly absorbing band at 273 nm (ε = 11.66 L·mol^−1^·cm^−1^, Figure S5) that we assigned to the n→π* transition of the acylhydrazine group (Figure 1C). In comparison, the UV–vis spectra of Boc‐**P1** showed a band at 290 nm (Figure 1C), in agreement with the cotton effect observed in the CD spectra of its chiral derivatives (Figure 1B). Moreover, the extinction coefficient (ε = 1693 L·mol^−1^·cm^−1^, Figure S6) at this wavelength was significantly higher than the one calculated for the monomer, in agreement with the presence of a chemical moiety that absorbs strongly in this region, such as the conjugated polymer backbone. Similarly, the UV–vis spectra of **P1** showed a band at 265 nm (Figure 1C), suggesting that conjugation for the fully deprotected polymer did not extend beyond 2–3 double bonds, as expected for *cis‐cisoid* or random coil conformations. The extinction coefficient (ε = 2091 L·mol^−1^·cm^−1^, Figure S7) was again significantly higher than the one calculated for the monomer. Finally, dynamic scanning calorimetry of **P1** revealed two main transitions at 135°C and 203°C (Figure 1D), consistent with those reported for other poly(acetylene)s with high *cis* content [74, 75].

The next stage in our project was to demonstrate that this reactive polymer **P1** could be easily functionalized under aqueous conditions to give polymers with a broad range of functionalities. Like for most of our previous work with poly(acryloyl hydrazide) [60, 63, 64, 65], **P1** was dissolved in a 100 mm aqueous solution of acetic acid, while the targeted aldehydes were dissolved in DMSO‐*d6*. These conditions ensured that we could investigate both hydrophilic and hydrophobic aldehydes. Experiments were performed with 1 equiv. of aldehyde per acyl hydrazine, and the degree of functionalization monitored by ^1^H‐NMR. A small library of aldehydes was investigated, including hydrophilic aldehydes such as 3‐guanidino‐N‐(3‐oxopropyl)propanamide (**4**) and d‐glucose (**5**), aliphatic aldehydes such as isovaleraldehyde (**6**) and octanal (**7**), aromatic aldehydes such as benzaldehyde (**8**), substituted aromatic aldehydes such as 2‐methylbenzaldehyde (**9**), 4‐hydroxybenzaldehyde (**10**) and 4‐fluorobenzaldehyde (**11**), and heterocyclic aldehydes such as imidazole‐4‐carboxyaldehyde (**12**), pyridine‐3‐carboxyaldehyde (**13**), indole‐3‐carboxyaldehyde (**14**) and uracil‐5‐carboxyaldehyde (**15**) (Table 1). This way, we were targeting the preparation of hydrophilic polymers (**P1‐*mod*‐4** and **P1‐*mod*‐5**), and polymers that should be soluble in water depending on the pH (**P1‐*mod*‐10**, **P1‐*mod*‐12,** and **P1‐*mod*‐13**). We also investigated the formation of hydrophobic polymers (reaction with aldehydes **6**–**9**, **11**, **14,** and **15**), because these moieties are prevalent in polymers with biomedical applications, such as in antimicrobial polymers [9, 10] or polymers for gene delivery [76, 77].

**TABLE 1:** Degree of functionalization of **P1** with 1 equiv. of different aldehydes.

Entry	Aldehyde	Loading	Entry	Aldehyde	Loading
P1‐*mod*‐4	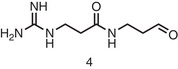	99%	P1‐*mod*‐10	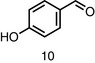	19.9%
P1‐*mod*‐5	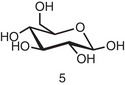	34.8%	P1‐*mod*‐11	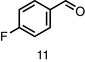	44.1%
P1‐*mod*‐6	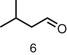	54.4%	P1‐*mod*‐12	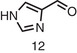	88.8%
P1‐*mod*‐7	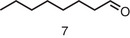	98.6%	P1‐*mod*‐13	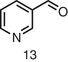	85.8%
P1‐*mod*‐8	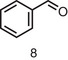	90.8%	P1‐*mod*‐14	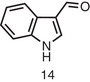	85.6%
P1‐*mod*‐9	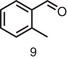	35.6%	P1‐*mod*‐15	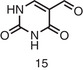	54.1%

All experiments were characterized after 24 h incubation at 30°C. Degree of functionalization calculated by ^1^H NMR.

Results were significantly different than those reported for poly(acryloyl hydrazide), for which steric hindrance seemed to be the dominating factor during the formation of the acyl hydrazones. In the case of our previous work, reaction with 1 equiv. of aldehyde resulted in ∼70% of functionalization for most aldehydes, with bulkier aldehydes normally having lower degrees of functionalization [61]. In contrast, for **P1,** we obtained almost quantitative degrees of functionalization for bulky aldehydes such as **4** and **7**, while other bulky aldehydes, such as **14,** gave a ∼30% increase in the degree of functionalization when compared to that reported for poly(acryloyl hydrazide) [61]. Heterocycles **12** and **13** gave similar very good degrees of functionalization, while reaction with uracil derivative **15** resulted in a moderate 54.1% of functionalization. A similar result was obtained for isovaleraldehyde (**6**), with only 54.4% functionalization. The worst results were obtained with substituted benzaldehyde derivatives **9**–**11**, in particular for 4‐hydroxybenzaldehyde (**10**), which only gave a 19.9% of functionalization. We believe this increase in the degree of functionalization for bulky aldehydes was related to the ability of the aldehydes to stabilize the helical conformation via side‐chain interactions, thus spreading this way the acyl hydrazines and making them more accessible. For instance, 3‐guanidino‐*N*‐(3‐oxopropyl)propanamide (**4**) could form H‐bonds via its amide residues, while octanal (**7**) and benzaldehyde (**8**) could stabilize the helical conformation via Van der Waals interactions. Moreover, the polymers formed with aldehydes **4**, **12,** and **13** would be mainly protonated under the conditions used for functionalization.^1^ This protonation would introduce positive charges on the polymer side‐chains that could improve the spatial orientation of the aldehyde and acyl hydrazine groups. Additionally, guanidinium ions can form like‐charge ion pairs [78], which have also been postulated for imidazolium cations [79]. Overall, the combination of hydrogen bonding, Van der Waals forces, and electrostatic repulsion could have a synergistic effect on the formation of the acyl hydrazones. These non‐covalent interactions are prevalent in the stabilization of poly(acetylene)s and other helical polymers [26, 66].

To probe this hypothesis, we performed another round of functionalization, using, in this case, hydrophobic aldehydes with an increasing number of carbons (Table 2). Many of these aldehydes should increase the non‐covalent interactions upon acyl hydrazone formation and stabilize the helix. This behavior was clearly the case for aliphatic aldehydes, for which going from octanal (**7**) to acetaldehyde (**16**) resulted in a dramatic decrease in the degree of functionalization from 98.6% to 17.6%. These values were in sharp contrast to those obtained for poly(acryloyl hydrazide), with a modest 62% obtained for octanal (**7**) while 89% was obtained for the smaller aldehyde **16** [61]. Moreover, the reaction of **P1** with 2‐ethylhexanal (**17**), which has the same number of carbons as octanal (**7**) but does not pack as efficiently due to branching [80], resulted in a significant decrease in the functionalization to 61.6%, highlighting again the role of non‐covalent interactions in the functionalization with these aldehydes.

**TABLE 2:** Degree of functionalization of **P1** with 1 equiv. of different aldehydes.

Entry	Aldehyde	Loading	Entry	Aldehyde	Loading
P1‐*mod*‐16	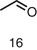	17.6%	P1‐*mod*‐18	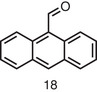	50.9%
P1‐*mod*‐6	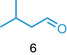	54.4%	P1‐*mod*‐19	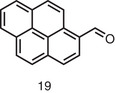	75.5%
P1‐*mod*‐17	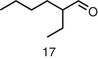	61.6%	P1‐*mod*‐9	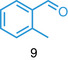	35.6%
P1‐*mod*‐7	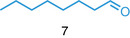	98.6%	P1‐*mod*‐20	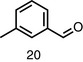	44.9%
P1‐*mod*‐8	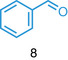	90.8%	P1‐*mod*‐21	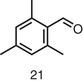	28.6%

All experiments were characterized after 24 h incubation at 30°C. Degree of functionalization calculated by ^1^H NMR. Entries in blue have been included in Table 1.

This stabilization of the helix due to non‐covalent interactions was not as clear for aromatic aldehydes (Table 2, aldehydes **8**, **18,** and **19**). In this case, the reaction of **P1** with anthracene derivative **18** resulted in a lower degree of functionalization (50.9%) than the benzene derivative **8**, while the reaction with pyrene derivative **19** resulted in an increase in functionalization to 75.5%, but still lower than for benzaldehyde (**8**). Moreover, substitution in the aromatic ring seemed to have a detrimental effect on the formation of the acyl hydrazones, as we obtained low degrees of functionalization for aldehydes **9**–**11** (Table 1). This decrease was also observed when the substituent was placed in a different position in the ring, like in the case of 3‐methylbenzadehyde (**20**), although the effect in this case was smaller than when the same substituent was placed in the ortho position (Table 2, **P1‐*mod*‐9** vs **P1‐*mod*‐20**). This effect seemed to be cumulative, and introducing multiple substituents in the same aromatic ring, as in the case of 2,4,6‐trimethylbenzaldehyde (**21**), resulted in a poor 28.6% of functionalization. We believe that the observed reduction in the degree of functionalization with these substituted benzaldehydes could be the result of both electronic and steric effects. Arrangement around a helical structure could disrupt the ideal packing of these aromatic moieties, which often involve off‐centered, tilted, or face‐to‐edge interactions [81, 82], thereby reducing this way the degree of functionalization.

Finally, we only observed a 34.8% of functionalization when **P1** was treated with 1 equiv. of d‐glucose (**5**) (Table 1). This degree of functionalization is not surprising since, in aqueous conditions, the equilibrium constant for the reaction of acyl hydrazines and monosaccharides is low [83]. A similar degree of functionalization was observed for other saccharides, including d‐mannose (**23**), d‐*N*‐acetyl‐glucosamine (**24**), and d‐lactose (**25**), with only d‐galactose (**22**) giving a lower degree of functionalization (Table 3).

**TABLE 3:** Degree of functionalization of **P1** with 1 equiv. of different carbohydrates.

Entry	Aldehyde	Loading	Entry	Aldehyde	Loading
P1‐*mod*‐22	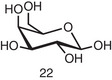	14.8%	P1‐*mod*‐24	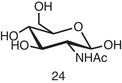	36.6%
P1‐*mod*‐23	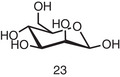	38.7%	P1‐*mod*‐25	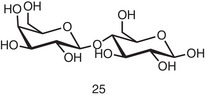	38.2%

All experiments were characterized after 24 h incubation at 30°C. Degree of functionalization calculated by ^1^H NMR.

**FIGURE 2:**
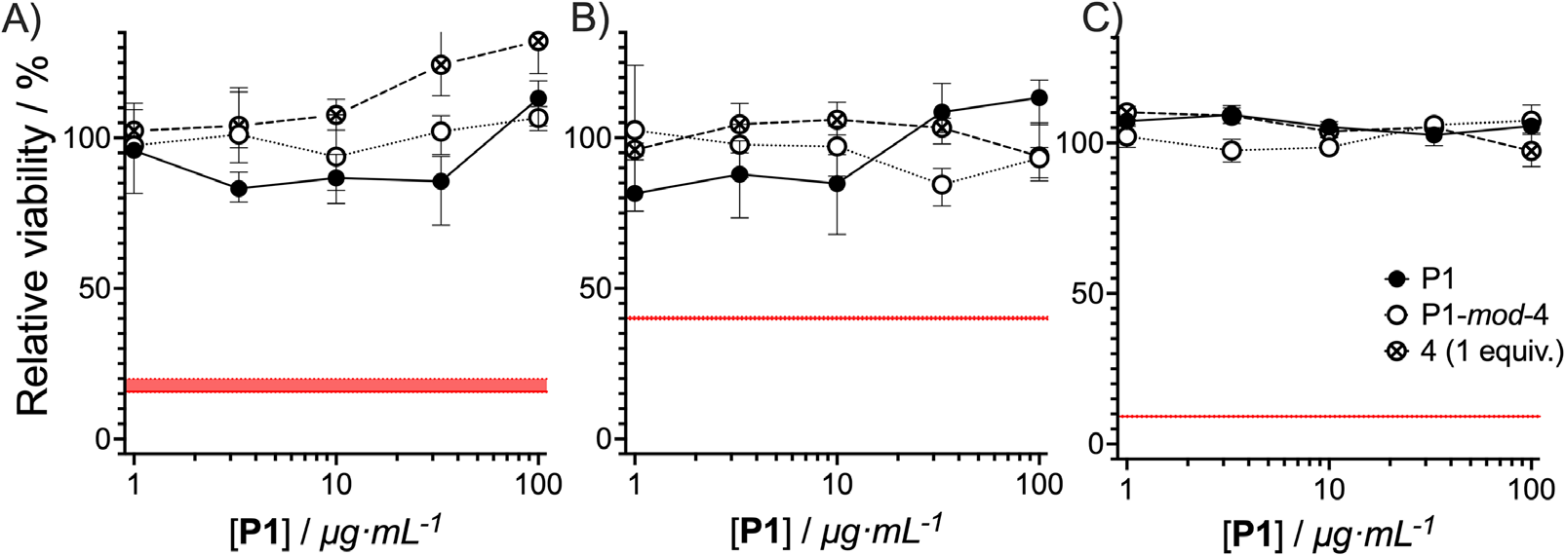
Relative viability of Hela (A), HEK 293T (B), and iMSC#9 (C) in the presence of **P1**, 3‐guanidino‐*N*‐(3‐oxopropyl)propanamide (**4**) and **P1‐*mod*‐4**. The data represent the average of 3 replicates. The error bars represent the range. Relative viability in the presence of an aqueous solution of 0.5% w/v of Triton is shown as a horizontal red line for each cell line.

Having demonstrated that **P1** could be readily functionalized with a range of aldehydes and that the degree of functionalization was dependent on the ability of each aldehyde to stabilize the helix conformation through non‐covalent interactions, the final stage of the work was to do a preliminary evaluation of the biocompatibility of this polymer. To this end, we measured the cytotoxicity of the polymer toward a small panel of cell lines, including HeLa, an immortalized cell line derived from cervical cancer cells, HEK 293T, another immortalized cell line derived from human embryonic kidney cells, and iMSC#9, immortalized mesenchymal stem cells derived from human bone marrow [84]. Cells were incubated in the absence and presence of **P1**, and the viability was monitored using CCK8, a water‐soluble formazan derivative that measures metabolic activity in cells. Results were normalized to untreated cells (Figure 2, 100%). Additionally, cells were incubated with **P1‐*mod*‐4** (Figure 2, 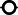), because 3‐guanidino‐*N*‐(3‐oxopropyl)propanamide (**4**) has been used prominently in our previous work to identify non‐viral vectors for gene delivery [60, 63, 64, 85]. Moreover, cationic polymers often exhibit toxicity toward cells [86]. Viability in the presence of **4** was also monitored to see if the toxicity observed could be associated with the excess of unreacted aldehyde (Figure 2, 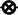). Finally, cells were also incubated with a 0.5% w/v aqueous solution of Triton X‐100 (Figure 2, red horizontal line), to compare the viability in the presence of the polymers against a known cytotoxic compound.

To our delight, neither **P1** nor **P1‐*mod*‐4** exhibited cytotoxicity against these cell lines across the range of concentrations tested (1‐100 µg·mL^−1^ in **P1**, concentration of aldehyde adjusted to give 1.4 equiv. per hydrazide). **P1** seemed to decrease the viability of HeLa and HEK 293T at low concentrations (≤33 µg·mL^−1^) but viabilities remained above 75% in all cases (Figures 2A,B, 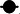). Moreover, guanidinium aldehyde **4** seemed to stress HeLa cells, leading to viabilities over 100% when cells were incubated with more than 10 µg·mL^−1^ of this compound (Figure 2A, 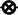). Remarkably, the conjugation of this aldehyde with **P1** removed that effect, with viabilities remaining above 85% for all the conditions tested with this functional polymer, **P1‐*mod*‐4**.

## 4 Conclusion

In conclusion, we have reported here the synthesis of a new reactive poly(acetylene), poly(*O*‐propargyl‐*N*‐amino carbamate) (**P1**), that can be easily functionalized under aqueous conditions to give functional poly(acetylene)s. **P1** can be prepared from commercially available alkynes using a straightforward synthetic protocol that includes polymerization with a Rh catalyst and deprotection under mild conditions. We have demonstrated that the formed polymer contains a high percentage of *cis* double bonds and that it may adopt a preferential *cis‐cisoid* conformation. This behavior is consistent with the CD observed for its chiral protected derivatives R‐Boc‐**P1** and S‐Boc‐**P1**, while a partially deprotected chiral polymer R‐P1* may adopt multiple conformations in aqueous solutions. We also demonstrate the functionalization of **P1** with a range of aldehydes, including cationic, aliphatic, aromatic, and heteroaromatic aldehydes, and a small set of saccharides. Higher degrees of functionalization were obtained for those aldehydes that could establish H‐bonds and other non‐covalent interactions, suggesting the stabilization of the helical conformation upon functionalization with these aldehydes. Finally, we demonstrate that **P1** and its cationic derivative **P1‐*mod*‐4** are non‐toxic to a small panel of cell lines. We believe the results presented herein demonstrate the potential of **P1** and related polymers to underpin the development of biomedical applications of poly(acetylene)s, an area currently underexplored.

## Author Contributions

T. L. performed M1, Boc‐P1, P1, R‐Boc‐P1, and S‐Boc‐P1 synthesis, P1‐*mod*‐X synthesis, ^1^H and ^13^C NMR, FT‐IR, UV‐vis, DSC, and CD characterization. G. B. performed M1, Boc‐P1, and P1 synthesis, kinetic experiments, and ^1^H‐NMR and UV‐vis characterization. D. C. performed M0, P0, M1, Boc‐P1, and P1 synthesis. A. D. performed R‐Boc‐P1 and R‐P1* synthesis, and CD characterization. A. R.‐B. performed aldehyde 4 and P1‐*mod*‐4 synthesis and assessment of cell viability experiments. A. R. performed aldehyde **4** synthesis. I. T.‐L. performed P1 synthesis. A.R.‐R. designed assessment of cell viability experiments and secure funding. P.F.‐T. conceived the project, secured funding, analyzed the data, and wrote the paper, with all other authors contributing to the final version of the manuscript.

## Conflicts of Interest

The authors declare no conflict of interest.

## Supporting information




**Supporting File**: marc70080‐sup‐0001‐SuppMat.pdf.

## Data Availability

The data that support the findings of this study are available from the corresponding author upon reasonable request.

## Footnotes

1 Simulations of the degree of protonation of decamers of the formed polymers, using Marvin Sketch pKa plugin (ChemAxon. MarvinSketch and Calculator Plugins (pKa Plugin), version 19.10.0; https://www.chemaxon.com), suggested that 97% of the pyridines would be protonated for **P1‐*mod*‐13**, while **P1‐*mod*‐12** and **P1‐*mod*‐4** would be fully protonated at pH 3.
