# Supplementary material for: Poly(O‐Propargyl‐N‐Amino Carbamate), a Reactive Polymer to Underpin Biomedical Applications of Poly(acetylene)s
Source: Macromol Rapid Commun. 2025 Oct 25;47(14):e00566. doi: 10.1002/marc.202500566 (PMC13384799; doi:10.1002/marc.202500566)
Supplement: Supplementary file 1 — Supporting File: marc70080‐sup‐0001‐SuppMat.pdf. [file MARC-47-e00566-s001.pdf]

Supporting Information

Poly(*O*-propargyl-*N*-amino carbamate), a reactive polymer to underpin biomedical applications of poly(acetylene)s.

Tom Leigh,[1] Greta Bellio,[2] Daniel Crisan,[1] Amit Deb,[1] Alba Ramil-Bouzas,[2,3] Andrey Romanyuk,[1] Ivan Torreiro-Leon,[2] Ana Rey-Rico,[3,4] Paco Fernandez-Trillo*,[1,2,4].*

[1] School of Chemistry, University of Birmingham, Edgbaston, Birmingham, UK, B15 2TT; [2] BioNanoChem Lab, Centro Interdisciplinar de Química e Bioloxía - CICA, Universidade da Coruña, 15071 A Coruña, Spain; [3] G-Cell, Centro Interdisciplinar de Química e Bioloxía - CICA, Universidade da Coruña, 15071 A Coruña, Spain; [4] Facultade de Ciencias, Universidade da Coruña, 15071 A Coruña,Spain.

f.ftrillo@udc.es

Table of Contents

1. Proposed synthesis of poly(propyloyl hydrazide) P0 2
2. Synthesis of poly(*O*-propargyl-*N*-amino carbamate) P1 and chiral derivatives 2
3. Calculation of extinction coefficient (ε) 3

1. Proposed synthesis of poly(propyloyl hydrazide) P0

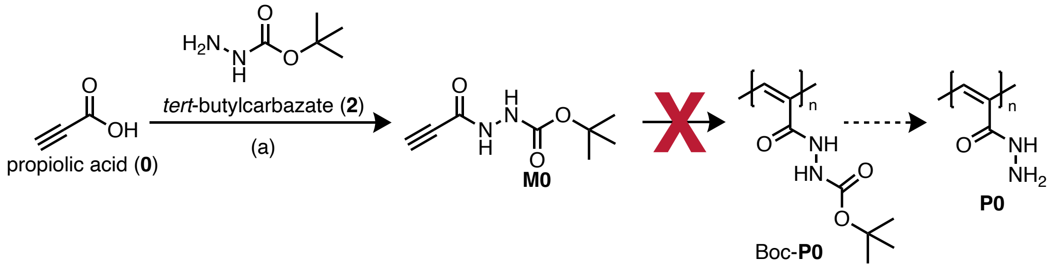

Figure S1 – Conditions: (a) (3-dimethylaminopropyl)carbodiimide hydrochloride, H_2O, rt, 15 min, 81%.

2. Synthesis of poly(*O*-propargyl-*N*-amino carbamate) P1 and chiral derivatives

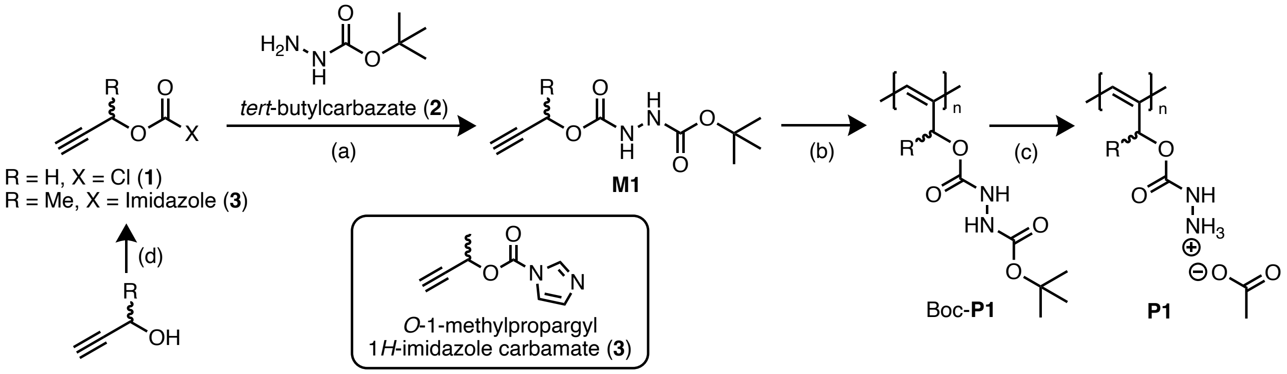

Figure S2 – Conditions: (a) ethyl acetate, 0 °C to rt. (b) [Rh(nbd)BPh_4], THF, 30 °C. (c) 1.- trifluoroacetic acid, rt. 2.- $NaHCO_3$, 0 °C to rt. 3.- 100 mM acetic acid in water, rt. (d) 1,1'-carbonyldiimidazole, ethyl acetate, rt.

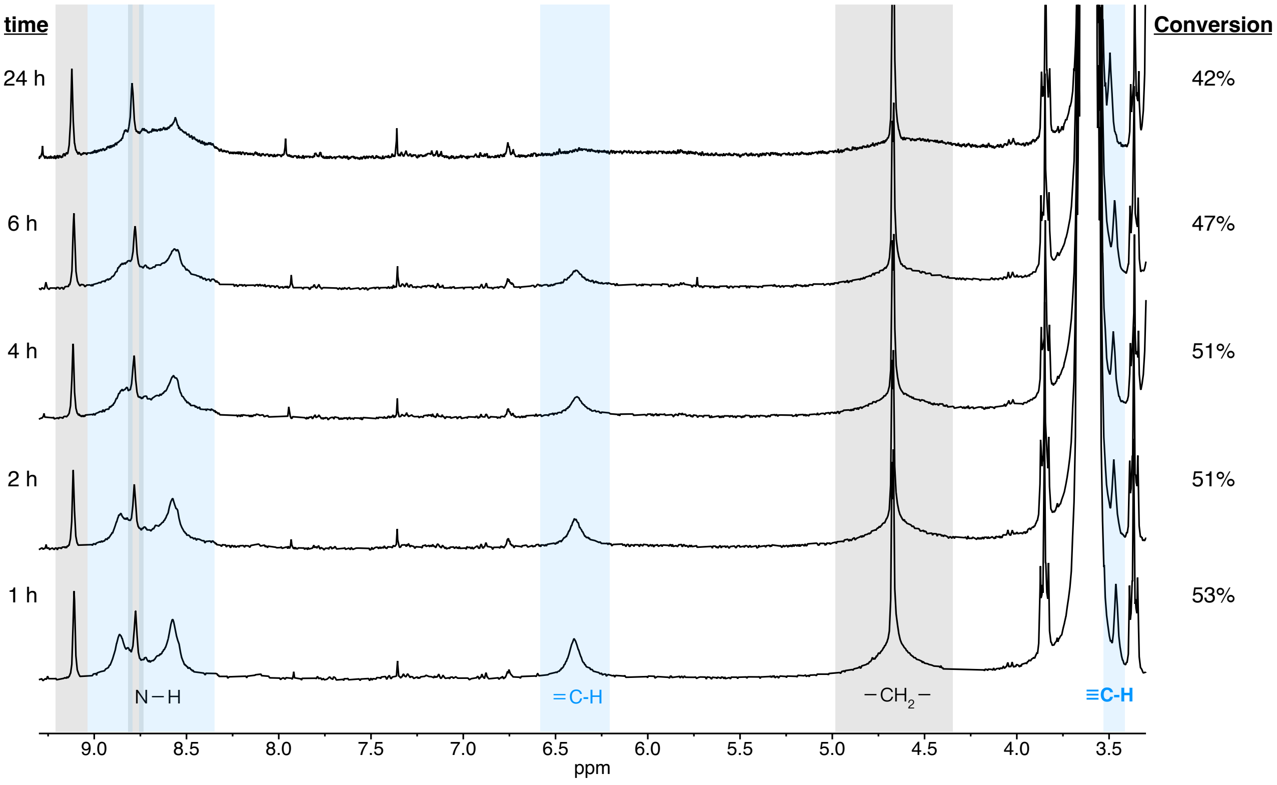

Figure S3 – ^1H NMR spectra of the polymerization of **M1** in the presence of [Rh(nbd)BPh_4] at different time intervals. Conversions are included for each time point.

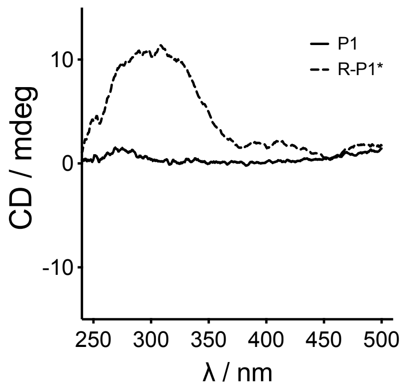

Figure S4 – CD spectra of **P1** (solid line) and partially deprotected R-**P1*** (dashed line) in 100 mM aqueous solution of acetic acid.

3. Calculation of extinction coefficient (ε)

A series of solutions with known concentrations of the analyte were prepared. UV-Vis spectra were recorded and their absorbance at the wavelength of maximum absorption (λ_{max}) calculated. This absorbance was plotted against the concentration (in M) of acylhydrazines in each analyte. ε was calculated using the following equation where l is the pathlength of the UV-Vis cuvette in cm.

$$\varepsilon \ / \ L \cdot mol^{-1} \cdot cm^{-1} = \frac{slope\ of\ Abs\ vs\ M}{l}$$

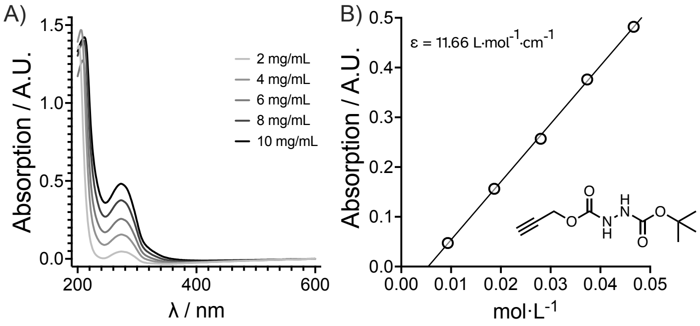

Figure S5 – A) UV-Vis spectra of **M1** in acetonitrile at different concentrations. B) Plot of absorbance at 273 nm as a function of **M1** concentration

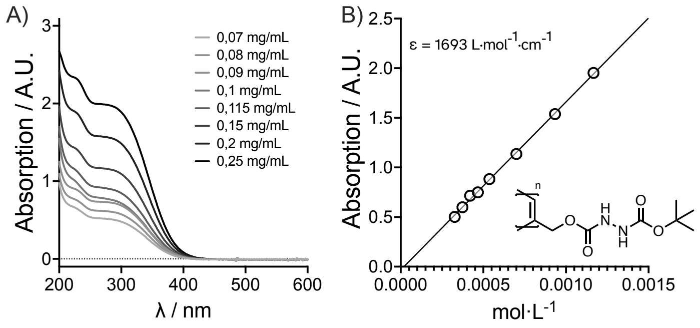

Figure S6 – A) UV-Vis spectra of Boc-**P1** in acetonitrile at different concentrations. B) Plot of absorbance at 290 nm as a function of **M1** concentration

poly(*O*-Propargyl-*N*-amino carbamate)

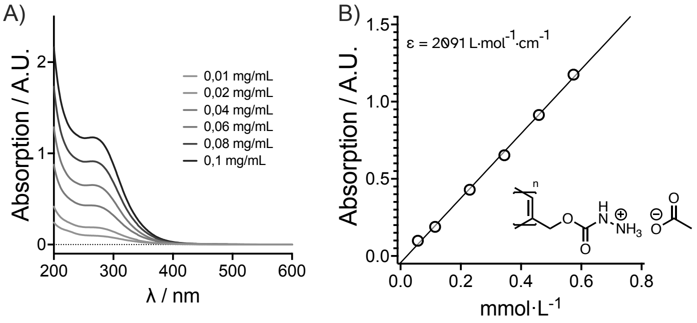

Figure S7 – A) UV-Vis spectra of **P1** in 100 mM aqueous solution of acetic acid at different concentrations. B) Plot of absorbance at 265 nm as a function of **M1** concentration
